# Deep Vein Thrombosis Provoked by Inferior Vena Cava Agenesis

**DOI:** 10.1155/2015/651436

**Published:** 2015-12-16

**Authors:** Raad A. Haddad, Mazin Saadaldin, Binay Kumar, Ghassan Bachuwa

**Affiliations:** Department of Internal Medicine, Michigan State University, Hurley Medical Center, 1 Hurley Plaza, Flint, MI 48503, USA

## Abstract

Inferior vena cava agenesis (IVCA) is a rare congenital anomaly that can be asymptomatic or present with vague, nonspecific symptoms, such as abdominal or lower back pain, or deep vein thrombosis (DVT). Here, we present a 55-year-old male who came with painless swelling and redness of his left lower limb. On examination, swelling and redness were noted extending from the left foot to the upper thigh; it was also warm compared to his right lower limb. Venous Doppler ultrasound was done which showed DVT extending up to the common femoral vein. Subsequently, computed-tomography (CT) of the chest and abdomen was done to exclude malignancy or venous flow obstruction; it revealed congenital absence (agenesis) of the infrarenal inferior vena cava (IVC).

## 1. Introduction

Inferior vena cava agenesis (IVCA) is a rare congenital anomaly. IVCA can be asymptomatic or can present with vague, nonspecific symptoms such as abdominal or lower back pain [1]. Most of the patients with IVCA can also present with DVT [2]. A clinical picture of a young healthy individual, usually in their second to fourth decades, with lower limb DVT without precipitating factors should raise the suspicion of IVCA [3].

## 2. Case Presentation

A 55-year-old male presented to the emergency department with left lower limb painless swelling and redness which started gradually over two weeks prior to presentation. He stated that he usually does not stay immobilized for a long period of time and there was no recent history of immobilization or surgeries. He denies any trauma to his left lower limb. His medical background includes obstructive sleep apnea. He has never been a smoker and his family history was negative for thromboembolic events or other hematological disorders. On physical examination, there were swelling and redness in his left lower limb extending from the foot to the upper thigh. Warmth was also noted compared to the right lower limb. The rest of the physical exam was unremarkable, there was no evidence of injury or venous stasis.

Biochemical and haematological investigations were normal. Venous Doppler ultrasound showed DVT extending from the common femoral vein at the level of the left groin to the popliteal vein at the level of the knee and a second DVT in the deep profunda vein.

**Figure 1:**
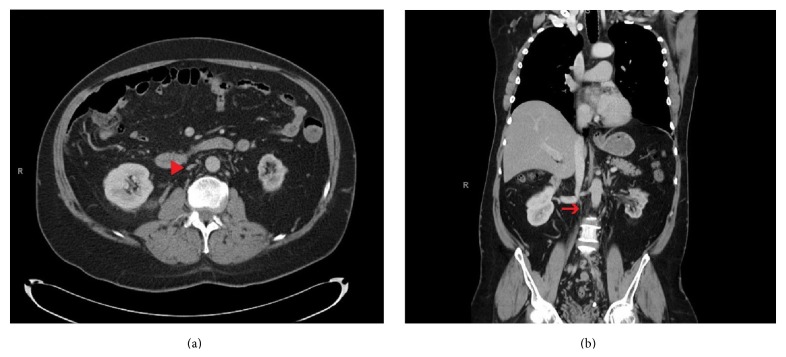
(a) Axial CT of the abdomen shows fibrosed infrarenal IVC (arrowhead). (b) Coronal CT of the abdomen shows thread-like fibrosis of the infrarenal IVC (arrow); note the presence of the IVC proximal to the renal veins.

Giving the extent of the thrombosis, CT of the chest and abdomen was considered to rule out further extension of the clot or suspected malignancy. CT revealed absent infrarenal IVC (Figure 1) associated with multiple para-aortic collaterals and tortuous dilatation of the inferior mesenteric vein. It also confirmed the presence of acute thrombosis involving the left external iliac and left common and superficial femoral veins.

Treatment was started initially with therapeutic dose of Enoxaparin. After that, he was started on Warfarin and required life-long anticoagulation. Following daily measurement of the calf and thigh circumference, his DVT had resolved. After discharge, life-long Warfarin was continued and he was advised to use elastic stocking. No recurrence of DVT has occurred at three and six months of follow-up. Thrombophilia studies, including lupus anticoagulant, cardiolipin antibody, antithrombin III, antinuclear antibody, factor V Leiden, and prothrombin gene mutation, were normal. However, his proteins C and S activity was low. As thrombophilia studies were done after initiating treatment, low activity level of anticoagulant proteins (proteins C and S) could be attributed to treatment with Warfarin [4].

## 3. Discussion

Congenital anomalies of the IVC are rare and commonly present as a lower limb DVT in young healthy adults; age presentation is variable but usually occurs with patients around their third decade of life [2, 5].

The exact aetiology of the IVCA remains unclear, but it is believed that thrombosis of the IVC in the perinatal period can lead to agenesis [6, 7]. Anomalies of the IVC are sometimes associated with other congenital anomalies, especially of the spleen and liver, as they have concurrent development with the IVC embryologically [1].

Diagnosis of IVCA is usually incidental, and once there is a suspicion, US, CT scan, MRI, or venogram of the abdomen are useful tools to detect IVCA or other congenital anomalies of the IVC [8]. Hypercoagulable studies are also recommended.

This anomaly may cause slow blood return through the collaterals leading to increased venous pressure, venous stasis, and thrombus formation [8–11]. Likelihood of developing pulmonary embolism (PE) is low; in a literature review of 62 cases by Lambert et al., 6 (9.67%) of the reported cases had PE that can be attributed to the travel of the clot through the dilated collateral veins that drains into the pulmonary venous vasculature [2, 12, 13].

Giving the rarity of the disease, there is no evidence based approach management [14]. Treatment is directed towards prevention of thrombosis formation or recurrence; life-long vitamin K antagonist is required for patients with IVCA in addition to elastic stocking and leg elevation to relieve venous stasis [1, 2, 8, 15]. Surgical correction can be an option for management depending on the severity of the venous stasis symptoms and response to medical and conservative treatment [11, 14, 16, 17].
